# Medication Use and Health Care Utilization After a Cost-sharing Increase in Schizophrenia: A Nationwide Analysis

**DOI:** 10.1097/MLR.0000000000001369

**Published:** 2020-07-23

**Authors:** Aleksi Hamina, Antti Tanskanen, Jari Tiihonen, Heidi Taipale

**Affiliations:** * School of Pharmacy, University of Eastern Finland, Kuopio, Finland; † Norwegian Centre for Addiction Research (SERAF) Institute of Clinical Medicine, University of Oslo, Oslo, Norway; ‡ Department of Forensic Psychiatry, Niuvanniemi Hospital, University of Eastern Finland, Kuopio; § Public Health Solutions, National Institute for Health and Welfare, Helsinki, Finland; ∥ Department of Clinical Neuroscience, Karolinska Institutet; ¶ Center for Psychiatric Research, Stockholm City Council, Stockholm, Sweden

**Keywords:** cost-sharing, health care utilization, interrupted time-series analysis, register-based, schizophrenia

## Abstract

**Background:**

Increases in prescription drug cost-sharing may decrease adherence to treatment among persons with schizophrenia and lead to discontinuation of use and an increased risk of hospitalization.

**Objective:**

The objective of this study was to investigate the impact of new deductible and increased drug copayments implemented on antipsychotic and other drug purchases and on rates of hospitalizations and primary care contacts among persons with schizophrenia in Finland.

**Research Design:**

Interrupted time series analysis.

**Subjects:**

All persons with schizophrenia in Finland who were alive at the beginning of 2015 (N=41,017).

**Measures:**

We measured the rates of antipsychotic, other psychotropic and cardiometabolic drug purchasers, hospitalizations, and primary care contacts during 2015 and 2016 with data collected from several nationwide health care registers.

**Results:**

During 2016, the proportion of antipsychotic purchasers decreased by −0.26 percentage points per month [95% confidence interval (CI): −0.47 to −0.05] compared with 2015. The trend of other psychotropic purchasers decreased to −0.13 percentage points per month in 2016 (95% CI: −0.22 to −0.04) compared with 2015 and cardiometabolic drug purchases to −0.17 percentage points per month (95% CI: −0.29 to −0.05) compared with 2015. The decreasing trend of psychiatric hospitalizations in 2015 halted in 2016. There were no other significant differences in health care utilization.

**Conclusions:**

In our nationwide time-series analysis, we observed decreases in the slopes of antipsychotic and other drug purchases of persons with schizophrenia after prescription drug cost-sharing increase implementation on January 1, 2016. Policymakers need to be aware of the unintended consequences of increasing cost-sharing among people with severe mental disorders.

## Abstract 2

Supplemental Digital Content is available in the text.

Schizophrenia is a chronic and debilitating illness defined by psychotic symptoms, but it also has a considerable impact on an individual’s behavior, cognition, and affect. The disease is often characterized by relapses, requiring intense treatment and hospitalization.^1^ Despite its relatively low prevalence, estimated at ∼0.3%, the global burden of schizophrenia is substantial.^2^ Persons with schizophrenia also very frequently present with comorbidities such as cardiovascular disease, diabetes, and substance use disorders, which have a high impact on an individual’s health and life expectancy.^3^–^5^ The direct and indirect costs of schizophrenia for individuals, health care systems, and societies are significant, and the lifetime costs can be as high as 1 million US$.^6^

Treatment with antipsychotic drugs is the cornerstone for managing the symptoms of schizophrenia. Because of the nature of the disease, persons with schizophrenia may exhibit poor adherence to treatment, which has been associated with worse outcomes, including a higher risk of relapse, rehospitalization, and suicide attempts.^7^–^9^ Decreased adherence to antipsychotic treatment is also likely to lead to increased hospital treatment costs.^10^–^12^ Increasing treatment adherence is consequently an important goal for treating patients with schizophrenia.^13^ Similarly, pharmacological treatment of comorbid conditions, such as cardiovascular and metabolic disorders, is considered highly important to reduce the burden of these diseases.^14^,^15^

In 2015, the Finnish government set a goal of EUR 150 million saving in public finances from 2017 directed at drug reimbursements (approximately US$ 163.1 by exchange rates in April 2020).^16^ Beginning in January 1, 2016, this included increases in cost-sharing of drug purchases, such as an introduction of annual initial deductible and increased drug copayments. The increases in cost-sharing were applied universally to all adults and all reimbursed prescription drugs in Finland, including drugs used for the treatment of schizophrenia and other mental disorders.

In US settings, increasing prescription copayments and other cost-containment strategies for persons with schizophrenia has previously been associated with worse compliance toward pharmacotherapy and increased psychiatric admissions.^17^–^21^ However, the effects in countries with national health care systems have been less frequently studied. Significant increases in psychiatric care copayments in the Netherlands have been associated with several negative outcomes among persons with schizophrenia, including increased involuntary commitment and higher downstream costs,^22^,^23^ but the effects of drug copayment increases have not been, to our knowledge, studied similarly. We aimed to investigate whether increased cost-sharing strategies had an impact on antipsychotic and other drug purchases and whether there were changes in the rates of hospitalizations and rates of primary care contacts among persons with schizophrenia.

## MATERIALS AND METHODS

### The Cost-sharing Intervention

In Finland, drug purchases from pharmacies are partly reimbursed for Finnish citizens by the Social Insurance Institution (SII). Drugs are dispensed for a maximum supply of 3 months at a time. The rate of drug reimbursement depends on the severity of the condition and the necessity of the drug, as defined by the SII. At a basic rate of reimbursement, the copayment is 60% of a drug’s price. For drugs used for chronic conditions, such as cardiovascular diseases, a decreased copayment can be applied, wherein the copayment is reduced to 35% of the drug’s price. For severe conditions such as schizophrenia, a fixed sum copayment is applied for each purchase, which increased from EUR 3 in 2015 to EUR 4.5 in 2016 (approximately US$ 3.3 to US$ 4.9). All reimbursements are applied in pharmacies, where the customer only pays the copayment.

In addition to increased cost-sharing for fixed copayment drugs, an initial deductible for all reimbursed drugs was introduced in 2016: an annual sum of EUR 50 (approximately US$ 54.3). Thus, beginning in January 2016, drug purchases were only reimbursed after exceeding the deductible sum. All purchases of reimbursable drugs count toward meeting the deductible, and it may take one or several purchases to exceed it. Children and adolescents are exempt from the deductible, as it is only applied the calendar year an individual turns 19. For a person with schizophrenia, for example, these changes may have increased the price of their first antipsychotic purchase of the year from EUR 3 in 2015 to EUR 54.5 in 2016, that is, the initial deductible plus the fixed copayment. This would result in an annual cost of EUR 12 in 2015 and EUR 68 (EUR 4.5 times 4+EUR 50) in 2016 for the first drug, if the person buys their medicine in 3-month supplies. Because the deductible is only paid once per year, the annual cost of 2 drugs would have been EUR 24 in 2015 and EUR 86 in 2016 (EUR 4.5 times 4 times 2+EUR 50). Both cost-sharing increases have stayed the same in Finland after their launch in 2016, but additional increases to antidiabetics were added in 2017.

### Study Population

We identified persons with schizophrenia from a national Hospital Discharge Register, which consists of all hospital care periods for the entire population of Finland since 1972.^24^ We extracted data on those discharged with a diagnosis of schizophrenia in 1972–2014 (N=61,889) utilizing International Classifiation of Diseases, 10th Revision (ICD-10) codes F20, F25; ICD-8, and ICD-9 codes 295.^25^ The Hospital Discharge Register has good coverage of persons with schizophrenia in Finland, and it has been utilized extensively for mental health previously.^24^ We excluded individuals aged under 19 years, as the initial deductible was not applied to those who are younger than 19. The final cohort consisted of 41,017 persons with schizophrenia, who were alive on the January 1, 2015.

### Drug Use

We collected data on drug use from a national Prescription Register, which consists of data on all reimbursed drugs purchased from Finnish pharmacies and is maintained by the SII. These data also include drugs an individual purchased before the EUR 50 initial deductible was fully met in 2016. Drugs in the Prescription Register are categorized according to the Anatomical Therapeutic Chemical system,^26^ which is a classification of pharmaceutical substances for drug utilization research and monitoring maintained by the World Health Organization. The system has five different levels, whereby each substance is classified into hierarchical groups according to their therapeutic use, pharmacology, and chemical structure.^26^ We defined antipsychotics as N05A, excluding lithium. Benzodiazepines and related drugs were defined as N05BA, N05CD, and N05CF, and antidepressants as N06A. Cardiometabolic drugs included insulins (A10A), oral antidiabetics (A10B), antihypertensives (C07–C09), and statins (C10AA). The register includes information on drug costs as total cost and as the cost for the SII, that is, the reimbursed sum. Drugs used during hospital care are not recorded in the register, as they are provided by the caring unit.

### Hospitalizations and Outpatient Primary Care Contacts

Data on hospitalizations during the follow-up was gathered from the Hospital Discharge Register. We collected data on all hospitalizations during 2015 and 2016 and categorized them according to the main diagnosis into psychiatric (ICD-10 codes F) and nonpsychiatric (all other disease codes).

In addition to hospitalizations, we analyzed changes in rates of outpatient primary care contacts and visits. We derived these data from a primary care register (Avohilmo), similarly maintained by the National Institute for Health and Welfare. This register consists of data from outpatient primary care records. Thus, these data include information on outpatient contacts with health care professionals, including in-person and telephone visits, as well as electronic appointments.

All persons in Finland have a unique identity number, which was utilized to link data from the different registers. This identifier was pseudonymized by the National Institute for Health and Welfare, before being transferred to the research team, and individual data were never analyzed separately. According to Finnish legislation, ethical committee approval is not required for register-based research wherein individual identifiers are removed or pseudonymized, and participants are not contacted in any way. The study obtained permissions for data use from register maintainers, the National Institute for Health and Welfare, and the Social Insurance Institution (SII).

### Statistical Analyses

We investigated new drug purchases each calendar month separately for 2015 and 2016, that is, the year before increases in cost-sharing and the year they were first implemented. The main interests were the copayment and the proportion of purchasers, that is, the percentage of the outpatient cohort buying prescription drugs per month. To examine whether there were any changes in drug use, we undertook interrupted time series analyses utilizing the proportions of purchasers each month. We chose this measure, as it shows the potential variation in purchasing behavior in relation to the size of the cohort at each timepoint. Interrupted time series analysis is a quasi-experimental design, which allows estimation of preintervention and postintervention trends after an intervention, for example, a policy change.^27^,^28^ The outcome, that is, the percentage of drug purchasers, is measured repeatedly (monthly) both before and after an intervention, thus strengthening a simple before-after analysis. The method accounts for pre-existing trends, such as an increase in the use of cardiometabolic drugs due to an aging cohort. Trend analysis of 2 years also considers possible seasonal variation, such as increasing purchases of drugs before holidays, for example, before New Year.

The effects of the copayment increase in the time series were estimated with segmented linear regression models, wherein we calculated regression coefficients for both 2015 and 2016. Possible autocorrelation of the timepoints was estimated utilizing a Durbin-Watson test.^27^ We performed all time-series analyses in autoregressive forms.

To describe the changes in cost-sharing, we calculated the average cost paid by the patients divided by person-years in the outpatient cohort each month. This number thus illustrates the cost of a drug class (eg, antipsychotics) during a year, were the copayment to stay at that level. We also calculated annual costs paid by the patient and annual total costs (ie, the sum of costs paid by the patient and the SII) for 2015 and 2016.

We also examined whether there were any changes in hospitalizations due to the copayment increase. Similarly to drug purchases, we analyzed the number of hospital admissions and outpatient contacts month-by-month in both 2015 and 2016, presented per 100 person-years. For outpatient analyses, we investigated the numbers of all outpatient contacts and in-person general practitioner contacts separately. This was carried out to prevent the large number of other contacts to dilute the possible effect on in-person general practitioner contacts, which we considered the most interesting measure. We then applied the segmented regression analysis to investigate changes in trends of hospitalization and outpatient contact rates from 2015 to 2016.

We completed descriptive analyses using percentages with 95% confidence intervals (CIs) and means with SDs. All statistical analyses were performed using the SAS statistical software (version 9.4; SAS Institute Inc., Cary, NC).

## RESULTS

Altogether 41,017 persons with schizophrenia were included in the analyses, and 2263 persons died during follow-up. Their mean age was 55 years (SD 15.1), and about half were men (50.2%) (Table 1). The mean number of years since the schizophrenia diagnosis was 21.2 (SD 12.4). At baseline (January 1, 2015), 78.9% had purchased antipsychotics in the previous 180 days (Table 1). Purchases of other psychotropic drugs and of cardiometabolic drugs were also frequent.

**TABLE 1:** Baseline Characteristics of the Cohort as of January 1, 2015

Characteristic	N (%)
Age (y)	
≤35	4741 (11.6)
36–55	14,574 (35.5)
>55	21,702 (52.9)
Age [mean (SD)] (y)	15.1 (55.1)
Year of diagnosis	
<1990	17,023 (41.5)
1990–1999	8446 (20.6)
2000–2009	10,846 (26.5)
2010–2014	4698 (11.5)
Male sex	20,591 (50.2)
Drug purchases in the past 180 d	
Antipsychotics	32,381 (78.9)
Other psychotropics	17,291 (42.2)
Antidepressants	10,653 (26.0)
Benzodiazepines and related drugs	10,873 (26.5)
Cardiometabolic drugs	19,082 (46.5)
Insulin	2116 (5.2)
Oral antidiabetics	7081 (17.3)
Antihypertensives	15,024 (36.6)
Statins	8318 (20.3)

**FIGURE 1:**
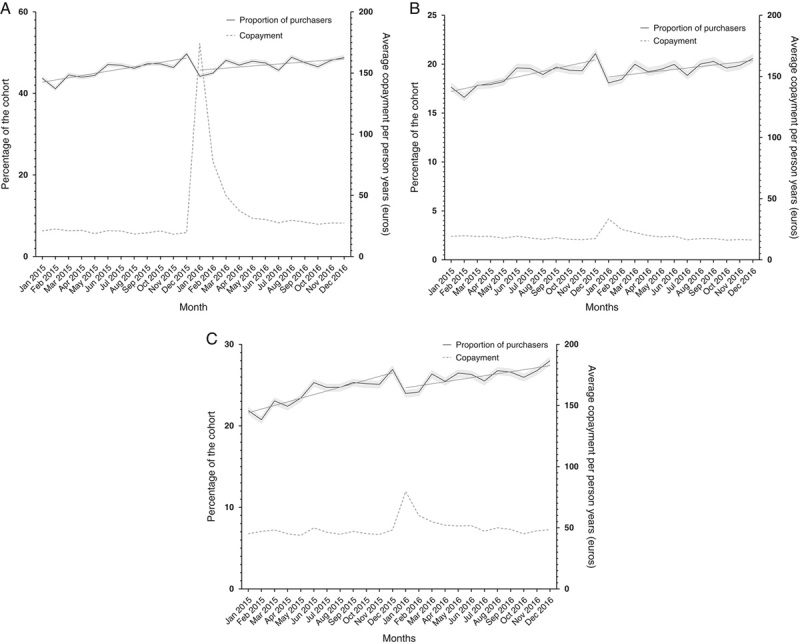
Trends in the proportion of purchasers of (A) antipsychotics; (B) benzodiazepines and related drugs and antidepressants; and (C) cardiometabolic drugs each month. The proportions of drug purchasers per month are displayed with 95% confidence intervals (left *y*-axis).

The monthly proportion of antipsychotic purchasers increased in 2015 from 43.7% in January (95% CI: 43.2–44.2) to 49.8% in December (95% CI: 49.2–50.2) (Fig. 1A). A similar increase was found in 2016, from 44.2% in January (95% CI: 43.7–44.7) to 48.8% in December (95% CI: 48.3–49.3). In segmented regression analyses, the proportion of antipsychotic purchasers increased by 0.55 percentage points per month in 2015 (95% CI: 0.39–0.71) (Table 2). In 2016, the proportion of purchasers increased by 0.29 percentage points per month in 2016 (95% CI: 0.09–0.49). There was a decrease in trend during 2016 of −0.26 percentage points per month (95% CI: −0.47 to −0.05) compared with 2015.

**TABLE 2:** Results of the Segmented Regression Analysis on Drug Use and Hospital Admissions Among Persons With Schizophrenia

Measure	2015 Slope	95% CI	Change in Slope in 2016	95% CI	2016 Slope	95% CI
Antipsychotic use	0.55	0.39–0.71	−0.26	−0.47 to −0.05	0.29	0.09–0.49
BZDR and antidepressant use	0.30	0.23–0.37	−0.13	−0.22 to −0.04	0.17	0.02–0.31
Cardiometabolic drug use	0.44	0.34–0.54	−0.17	−0.29 to −0.05	0.27	0.07–0.46
Psychiatric hospitalizations	−0.33	−0.57 to −0.10	0.18	−0.15 to 0.51	−0.15	−0.62 to 0.86
Nonpsychiatric hospitalizations	−0.20	−0.61 to 0.22	0.11	−0.47 to 0.70	−0.08	−0.65 to 0.48
Outpatient health care contacts	0.06	−0.11 to 0.22	0.00	−0.23 to 0.24	0.06	−0.31 to 0.43
Outpatient general practitioner visits	0.00	−0.04 to 0.05	−0.01	−0.07 to 0.06	−0.00	−0.11 to 0.10

The slope presents change as percentage points per month.

BZDR indicates benzodiazepines and related drugs; CI, confidence interval.

As expected, the average copayment of antipsychotics per person-years increased sharply in early 2016 (Fig. 1A). The annual copayment per person-years of antipsychotics increased from EUR 20.3 in 2015 to EUR 47.5 in 2016. However, the total annual cost of antipsychotics per person decreased from EUR 1042.4 in 2015 to EUR 964.8 in 2016.

Similarly to antipsychotics, the proportion of purchasers of benzodiazepines and related drugs and antidepressants increased in 2015 from 17.7% in January (95% CI: 17.3–18.0) to 21.1% in December (95% CI: 20.7–21.5) and in 2016 from 18.1% in January (95% CI: 17.7–18.5) to 20.6% in December (95% CI: 20.2–21.0) (Fig. 1B). The increase was 0.3 percentage points per month (95% CI: 0.23–0.37) (Table 2) in 2015. The slope increased 0.17 percentage points per month (95% CI 0.02–0.31) in 2016, a decrease of −0.13 percentage points per month compared with 2015 (95% CI: −0.22 to −0.04). The annual copayment per person-years somewhat increased, from EUR 18.1 in 2015 to EUR 19.9 in 2016.

The proportion of cardiometabolic drug purchasers increased in 2015 from 21.9% in January (95% CI: 21.5–22.3) to 26.9% in December (95% CI: 26.5–27.4) and in 2016 from 24.0% in January (95% CI: 23.6–24.4) to 28.0% in December (95% CI: 27.5–28.4) (Fig. 1C). The trend was an increase of 0.44 percentage points per month in 2015 (95% CI: 0.34–0.54) and an increase by 0.27 percentage points per month in 2016 (95% CI: 0.07–0.46). This was a decrease in the slope of −0.17 percentage points per month in 2016 (95% CI: −0.29 to −0.05) compared with 2015. The annual copayment per person-years for cardiometabolic drugs increased from EUR 46.3 in 2015 to EUR 53.1 in 2016.

**FIGURE 2:**
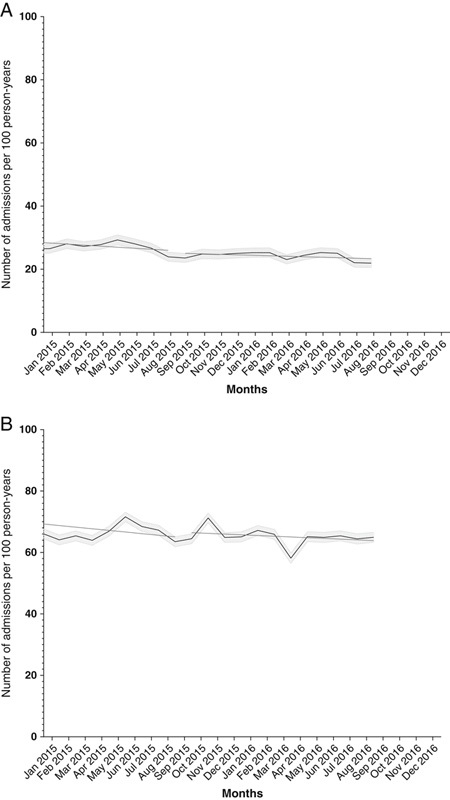
Trends in (A) psychiatric and (B) nonpsychiatric hospital admissions before and after cost-sharing increase (beginning of 2016). Rates are displayed with 95% confidence intervals.

As for hospital care, psychiatric admissions were decreasing in 2015 (−0.33 percentage points per month, 95% CI −0.57 to −0.10) (Fig. 2A, Table 2). This trend stabilized in 2016. However, the change in trend was nonsignificant in 2016 compared with 2015 (Table 2). There were no significant changes in trends of nonpsychiatric admissions before or after the cost-sharing increase (Fig. 2B, Table 2). As for the outpatient primary care contacts and general practitioner visits, the rates remained similar from 2015 to 2016 (Supplement Fig. 1, Supplemental Digital Content 1, http://links.lww.com/MLR/C42). There were no significant changes in these trends between the 2 years (Table 2).

## DISCUSSION

In our nationwide analysis of persons with schizophrenia, we found a decrease in the trends of drug purchasers after cost-sharing increases in Finland in 2016. The changes in trends were somewhat modest, but our results are consistent with previous studies on the subject.^17^–^19^,^29^ Moreover, a decreasing trend of psychiatric hospitalizations in 2015 halted in 2016, although the change in trend was not significant. The policy change did not significantly change other trends of hospital admissions or rates of primary care contacts. To our knowledge, this is the first nationwide study on the subject and the first to investigate changes in drug purchases after cost-sharing increases in a country with universal health care.

Our results on the decreases in the slopes of drug use after cost-sharing increases among persons with schizophrenia are in line with previous research from the United States.^17^–^19^,^29^ Schizophrenia frequently leads to work disability, and the majority of patients are on a disability pension in Finland.^30^ Increasing prescription costs for low-income individuals can, therefore, be expected to decrease drug use, regardless of stronger financial support systems in Finland compared with the United States. Moreover, even relatively small increases in prescription copayments have been previously found to decrease drug use among persons with schizophrenia.^29^ Especially, perceived copayment burden has been associated with decreased adherence and poorer outcomes in schizophrenia.^31^ However, we did not find larger decreases in the purchases of nonantipsychotic drugs, which would indicate prioritizing, as previously reported by Doshi and colleagues. Whether prioritizing of drug purchases can be detected after austerity measures in other countries with universal health care could be an area for future research.

We did not find significant changes in the trends of hospitalizations or in primary care utilization in this study. However, the decreasing trend of psychiatric hospitalizations did end in 2016, after the increase in prescription drug cost-sharing. This indicates small increases in psychiatric hospitalizations in 2016 compared with what would have been expected, but overall these changes were small. This may be due to relatively small changes in the rates of drug purchasers. As for nonpsychiatric admissions, the effects of decreased cardiometabolic drug adherence may also require a longer lag-time, that is, possibly several years to realize. Pharmacotherapy of conditions such as diabetes and cardiovascular diseases are important interventions in reducing the health disparities between persons with schizophrenia and the general population.^5^,^14^,^32^ Although life expectancy in persons with schizophrenia in Finland has been improving at a similar rate as among those without the disease, the disparity between these populations has remained the same.^4^ Increasing adherence to cardiometabolic drugs thus remains an important goal for health policymakers, as well. Future studies should investigate the long-term effects of cost-sharing policies on health, mortality, and long-term admission rates in this population.

Our results have important implications for both policymakers and health care professionals treating patients with schizophrenia. First, policymakers need to be aware of the potential detrimental impact of increased prescription drug cost-sharing strategies. Consideration is required as to whether people with severe illnesses should be excluded from such programs. Second, health care professionals should consider the economic effects of cost-containment strategies on their individual patients to ensure adherence to treatment is not compromised. Actively delivering information to patients on available resources, such as social safety nets, could help reduce the most negative effects of such policies on health.

### Strengths and Limitations

A major strength of this study was its nationwide coverage of the utilized databases. Our analyses included all individuals who had been hospitalized due to schizophrenia and were alive in Finland at the beginning of 2015. Similarly, the Prescription Register includes all purchases of reimbursed drugs during the study period. It should be noted that there are some drugs or package sizes of drugs that were not reimbursed. These represent a small minority of available drugs, however, and are very unlikely to have an effect on our results. Moreover, we were able to analyze both the rates of hospitalizations and primary care contacts that can together be considered a very good estimation of health care utilization in this population. Our results are likely to be generalizable to other high-income countries with universal health care.

Our main method, the interrupted time series analysis, is a strong quasi-experimental design on investigating the effects of policy change over time.^28^ Measuring change in slope considers pre-existing trends, such as the increasing drug purchase rates in our cohort. A limitation of our study is the absence of a control group, that is, of a similar cohort that is not affected by the policy change. Other, yet simultaneous, changes could have thus affected drug prescribing or purchasing in 2016. We are not aware of any such changes, however. Similarly, as both of the new cost-sharing interventions were applied simultaneously, it was not possible to analyze the effect of either one individually. It is also possible that the cost-sharing increases would have had a different effect on those diagnosed with schizophrenia very recently or on those who have never been hospitalized for their schizophrenia. Unfortunately, we were not able to include people diagnosed after 2014 or never hospitalized patients to this study. In addition, the follow-up period of 1 year after the intervention may be too short to observe changes in especially nonpsychiatric hospitalizations. Because of the reoccurring nature of the annual deductible, we could not lengthen the postintervention follow-up period to subsequent years. Finally, measuring purchases from health care registries results in rough estimates of actual prescription drug use. Our measure of proportion of purchasers per month may be sensitive to reductions in numbers of drugs in use and reductions in dose, in addition to absolute termination of drug use, but there may be cases wherein we were unable to measure changes in drug purchasing behaviors.

## CONCLUSIONS

In our nationwide analysis, a prescription drug cost-sharing increase in 2016 in Finland decreased the trends of drug purchases among persons with schizophrenia. A decreasing trend of psychiatric hospitalizations in 2015 halted in 2016, but the change in trend was nonsignificant. These results indicate that drug purchasing behavior was affected by the increase in cost-sharing. Whether health-related outcomes are affected in long-term follow-up should be a topic for future research. Policymakers should be aware of the potential effects of austerity measures on vulnerable populations, such as those with severe mental disorders.

## Supplementary Material

SUPPLEMENTARY MATERIAL

Supplemental Digital Content is available for this article. Direct URL citations appear in the printed text and are provided in the HTML and PDF versions of this article on the journal's website, www.lww-medicalcare.com.
