# Supplementary material for: Medication Use and Health Care Utilization After a Cost-sharing Increase in Schizophrenia: A Nationwide Analysis
Source: Med Care. 2020 Jul 23;58(9):763–9. doi: 10.1097/MLR.0000000000001369 (PMC7497420; doi:10.1097/MLR.0000000000001369)
Supplement: SUPPLEMENTARY MATERIAL [file mlr-58-763-s001.pdf]

a)

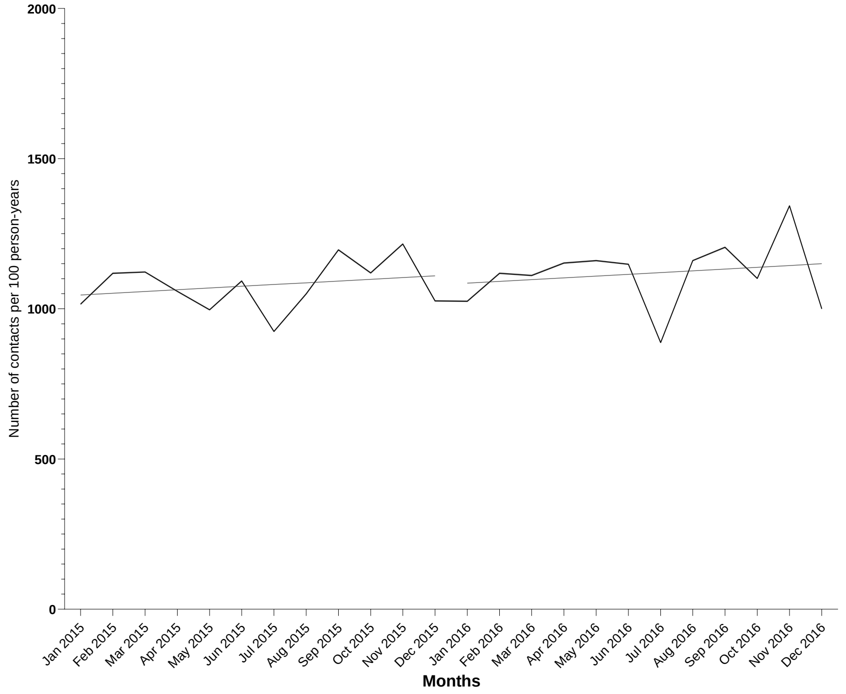

b)

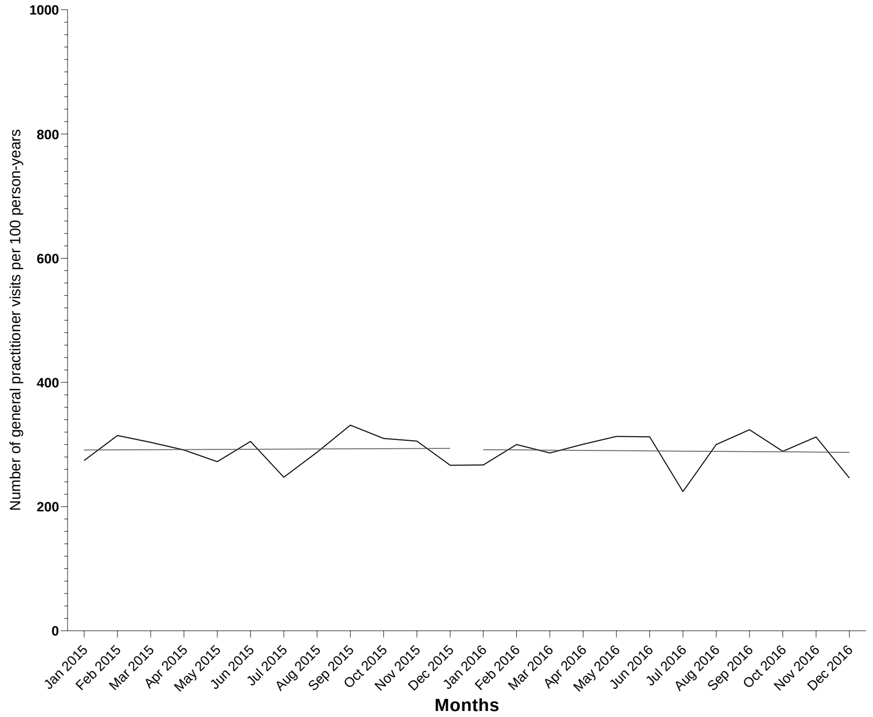

Supplemental Figure 1. Trends in a) all outpatient primary care contacts and in b) outpatient primary care general practitioner visits before and after cost-sharing increase (beginning of 2016). Rates are displayed with 95% confidence intervals.
